# Convention of College Faculties

**Published:** 1895-08

**Authors:** 


### Convention of College Faculties.

We take great pleasure in publishing this month an outlined programme of the coming meeting of the Association of Faculties of North America. All of the subjects to be discussed are of very great importance in this laudable work, and the selected speakers are so well known in the circle of veterinary teachers that we could wish that the sessions would be open ones, that all might have the pleasure of listening to the consideration of these subjects.

